# Lethality of mice bearing a knockout of the *Ngly1*-gene is partially rescued by the additional deletion of the *Engase* gene

**DOI:** 10.1371/journal.pgen.1006696

**Published:** 2017-04-20

**Authors:** Haruhiko Fujihira, Yuki Masahara-Negishi, Masaru Tamura, Chengcheng Huang, Yoichiro Harada, Shigeharu Wakana, Daisuke Takakura, Nana Kawasaki, Naoyuki Taniguchi, Gen Kondoh, Tadashi Yamashita, Yoko Funakoshi, Tadashi Suzuki

**Affiliations:** 1 Glycometabolome Team, Systems Glycobiology Research Group, RIKEN-Max Planck Joint Research Center, Global Research Cluster, RIKEN, Saitama, Japan; 2 Technology and Development Team for Mouse Phenotype Analysis, Japan Mouse Clinic, BioResourse Center, RIKEN, Ibaraki, Japan; 3 Graduate School of Medical Life Science, Yokohama City University, Kanagawa, Japan; 4 Disease Glycomics Team, Systems Glycobiology Research Group, RIKEN-Max Planck Joint Research Center, Global Research Cluster, RIKEN, Saitama, Japan; 5 Laboratory of Integrative Biological Science, Institute for Frontier Life and Medical Sciences, Kyoto University, Kyoto, Japan; 6 Laboratory of Biochemistry, School of Veterinary Medicine, Azabu University, Kanagawa, Japan; University of Georgia, UNITED STATES

## Abstract

The cytoplasmic peptide:*N*-glycanase (Ngly1 in mammals) is a de-*N*-glycosylating enzyme that is highly conserved among eukaryotes. It was recently reported that subjects harboring mutations in the *NGLY1* gene exhibited severe systemic symptoms (*NGLY1*-deficiency). While the enzyme obviously has a critical role in mammals, its precise function remains unclear. In this study, we analyzed *Ngly1*-deficient mice and found that they are embryonic lethal in C57BL/6 background. Surprisingly, the additional deletion of the gene encoding endo-β-*N*-acetylglucosaminidase (*Engase*), which is another de-*N*-glycosylating enzyme but leaves a single GlcNAc at glycosylated Asn residues, resulted in the partial rescue of the lethality of the *Ngly1*-deficient mice. Additionally, we also found that a change in the genetic background of C57BL/6 mice, produced by crossing the mice with an outbred mouse strain (ICR) could partially rescue the embryonic lethality of *Ngly1*-deficient mice. Viable *Ngly1*-deficient mice in a C57BL/6 and ICR mixed background, however, showed a very severe phenotype reminiscent of the symptoms of *NGLY1*-deficiency subjects. Again, many of those defects were strongly suppressed by the additional deletion of *Engase* in the C57BL/6 and ICR mixed background. The defects observed in *Ngly1/Engase*-deficient mice (C57BL/6 background) and *Ngly1*-deficient mice (C57BL/6 and ICR mixed background) closely resembled some of the symptoms of patients with an *NGLY1*-deficiency. These observations strongly suggest that the *Ngly1*- or *Ngly1/Engase*-deficient mice could serve as a valuable animal model for studies related to the pathogenesis of the *NGLY1-*deficiency, and that cytoplasmic ENGase represents one of the potential therapeutic targets for this genetic disorder.

## Author summary

Ngly1 is a cytoplasmic de-*N*-glycosylating enzyme that is ubiquitously found in eukaryotes. This enzyme is involved in a process referred to as endoplasmic reticulum-associated degradation (ERAD), one of the quality control mechanisms for newly synthesized proteins. A genetic disorder, *NGLY1*-deficiency, caused by mutations in the *NGLY1* gene has recently been discovered. However, the precise mechanism for the pathogenesis of this devastating disease continues to remain unclear. We report herein that *Ngly1*-deficient mice are embryonically lethal in a C57BL/6 background. Surprisingly, the lethality was suppressed by crossing the mice with an outbred mouse strain (ICR), suggesting that the phenotypic consequence of Ngly1 is greatly influenced by their genetic background. In both cases, the additional deletion of *Engase* in *Ngly1*-deficient mice could strongly mitigate the phenotypes. Interestingly, the remaining defects in *Ngly1*-deficient or *Ngly1*/*Engase*-deficient mice were reminiscent of the symptoms of subjects with an *NGLY1*-deficiency. Our results clearly point to the importance of Ngly1 in mammals and show that the inhibition of ENGase represents an effective therapy for treating an *NGLY1*-deficiency. Most importantly, the mice described herein could serve as valuable viable model mice for studies related to the pathophysiology of an *NGLY1*-deficiency.

## Introduction

**Fig 1:**
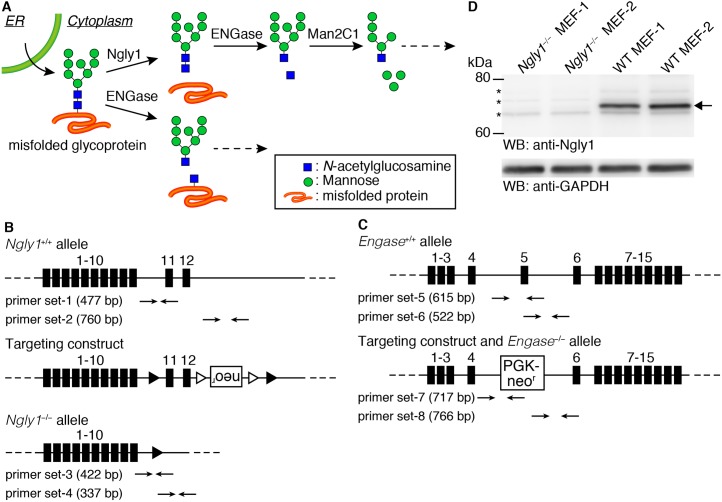
Generation of *Ngly1*- and *Engase*-deficient mice. **(A)** Schematic views of the non-lysosomal degradation of free oligosaccharides (upper scheme) and the action of ENGase on glycoproteins (lower scheme). **(B, C)** A diagram of the targeting construct used to generate *Ngly1*-deficient mice (B) and *Engase*-deficient mice (C) (Figures Reproduced from ref. 27). In the *Ngly1*^*−/−*^ allele, from exon 11 to 12 were flanked by loxP site (filled triangles). Open triangles indicate FRT sites. In *Engase*^*−/−*^ allele, exon 5 was replaced with a PGK-Neo^r^ cassette. **(D)** Cytoplasmic fraction of MEF cells derived from *Ngly1*^*−/−*^ or *Ngly1*^*+/+*^ (wild type, WT) embryos were immunoblotted with an antibody against mouse Ngly1. GAPDH was used as a loading control. Allow indicates Ngly1 and asterisks indicate non-specific bands.

The endoplasmic reticulum (ER) is the organelle responsible for the biosynthesis of proteins that pass through the secretory pathway. This organelle has an efficient protein quality control or homeostasis system, in which abnormal or excess proteins are targeted for destruction [1–3]. In such systems, cytoplasmic proteasomes play a major role in degrading proteins, and the degradation system is often referred to as ER-associated degradation, or ERAD in short. Ngly1 is a highly conserved deglycosylating enzyme that is involved in the ERAD process [4–6]. Ngly1 cleaves *N*-glycans from misfolded glycoproteins during the ERAD process, and is thought to play an important role in the efficient degradation of misfolded glycoproteins [7–11]. *N*-glycans released by Ngly1 are known to be processed by cytoplasmic glycosidases such as ENGase and the α-mannosidase, Man2C1 (Fig 1A, upper scheme) [12–14]. Cytoplasmic ENGase is another deglycosylating enzyme that acts directly on *N-*glycans that are attached to glycoproteins but leaves a single *N*-acetylglucosamine (GlcNAc) residue attached to the protein (Fig 1A, lower scheme). This enzyme belongs to the Glycosyl Hydrolase (GH) family 85, and while conserved widely in eukaryotes, some yeast, including *S*. *cerevisiae* and *S*. *pombe*, do not produce this enzyme.

Several studies dealing with the biological functions of Ngly1 and/or ENGase have been reported. Mutants of *Ngly1* and its orthologues have been analyzed in various organisms [7, 15–19]. Interestingly, the phenotypic consequences were found to be quite distinct between species; for example, budding yeast show no significant viability/growth defects [7], while mutant flies exhibited severe growth delay/arrest [15]. The molecular details behind the phenotypes, however, remain poorly understood. As of this writing, mutants of *Engase* have been analyzed in *A*. *thaliana* [20] and *C*. *elegans* [21] but, in both cases, no significant phenotypes were observed.

In 2012, the first patient harboring mutations in *NGLY1* alleles (*NGLY1-*deficiency) was identified through an exome analysis [22] and, since then, clinical cases involving humans have been reported [23–26]. These patients show very severe systemic symptoms such as delayed global development, movement disorders, hypotonia and hypo/alacrima [24, 26]. These reports point to the biological significance of Ngly1 in the normal development of mammals. More recently, using a model ERAD substrate, we reported that the ablation of *Ngly1* causes a disruption in the ERAD process in mouse embryonic fibroblast (MEF) cells [27]. Interestingly, this ERAD disruption was found to be caused by an unexpected deglycosylating activity of ENGase, and the direct action of this enzyme towards the model substrate was shown to result in the formation of aggregation-prone *N-*GlcNAc proteins [27]. Moreover, the disruption of ERAD in *Ngly1*-deficient cells was restored by the additional deletion of the *Engase* gene. While this result using a model substrate suggests that an ENGase inhibitor could be a potential therapeutic target for treating an *NGLY1*-deficiency, the issue of how *Engase*-deletion affects mice phenotypes lacking *Ngly1* remains unknown.

## Results

### *Ngly1*^*−/−*^ mice in C57BL/6 are embryonically lethal

The goal of this study was to clarify the details of the biological function of the deglycosylating enzymes, Ngly1 and ENGase, at the individual level in mice. To this end, we used mice (C57BL/6 background mice) that had been generated in a previous study, in which the *Ngly1* and *Engase* genes had been knocked out [27]. The knockout constructs of *Ngly1* and *Engase* are shown in Fig 1B and 1C, respectively. While the generation of these KO mice has been described previously [27], a detailed phenotypic analysis of those mice has not been reported. The targeted genomic disruption of Ngly1 and ENGase was confirmed by PCR using 8 sets of primers (Fig 1B and 1C; S1 Table for primer sequences). The loss of Ngly1 activity was confirmed by an activity assay [27]. We further examined the expression of the Ngly1 protein by western blot analysis using cytoplasmic fractions from MEF cells. As shown in Fig 1D, the loss of Ngly1 in MEF cells from *Ngly1*^*−/−*^ mice was confirmed. In the case of ENGase, we confirmed the loss of the ENGase activity through an activity assay in a previous study [27] as well as by conducting a detailed structural analysis of the free oligosaccharides (fOSs) in the cytoplasm of MEF cells [28]. The *Ngly1* heterozygous (*Ngly1*^*−/+*^) mice were fertile and did not show any obviously recognizable phenotypes. However, viable homozygous *Ngly1*^*−/−*^ pups were not produced, despite the repeated crossing of the *Ngly1*^*−/+*^ mice (Table 1), suggesting that the deletion of the *Ngly1* allele results in a lethal condition in C57BL/6 mice. To delineate the timing of the lethality, *Ngly1*^*−/+*^ mice were crossed and embryos were collected at several stages of gestation. The viability of collected embryos was confirmed by checking their heart beat and their genotypes were analyzed using genomic DNA extracted from the amnion. The results of this genotyping are summarized in Table 1. The *Ngly1*^*−/−*^ embryos were viable, even at embryonic day 18.5 (E18.5), one day prior to birth. At the same time, however, about 30% of *Ngly1*^*−/−*^ embryos were inviable at later stages of development (E17.5–18.5). When embryos were collected early in the morning of the day of birth, only the *Ngly1*^*−/+*^ and *Ngly1*^*+/+*^ embryos were alive when we revived them by gentle massaging, but no *Ngly1*^*−/−*^ mice could be revived. We also analyzed the genotype of pups within a few hours after their birth (P0) and confirmed the absence of *Ngly1*^*−/−*^ pups (Table 1). At E14.5 and E16.5, however, only viable *Ngly1*^*−/−*^ embryos were observed. Therefore, the lethality caused by the Ngly1 deficiency appears to occur between E16.5 and before birth.

**Table 1:** Results of the genotyping of pups/embryos by crossing of *Ngly1*^*−/+*^, or *Ngly1*^*−/+*^*;Engase*^*−/−*^ mice in the C57BL/6 background.

Stage	Genotype	Number of viable embryos/pups	Number of inviable embryos/pups
E14.5	*Ngly1*^+/+^	**18** (21.95) [25.00]	**0**
	*Ngly1*^−/+^	**50** (60.98) [50.00]	**0**
	*Ngly1*^−/−^	**14** (17.07) [25.00]	**0**
E16.5	*Ngly1*^+/+^	**56** (28.00) [25.00]	**0**
	*Ngly1*^−/+^	**113** (56.50) [50.00]	**0**
	*Ngly1*^−/−^	**31** (15.50) [25.00]	**0**
	*Ngly1*^+/+^;*Engase*^−/−^	**20** (22.73) [25.00]	**0**
	*Ngly1*^−/+^;*Engase*^−/−^	**52** (59.09) [50.00]	**0**
	*Ngly1*^−/−^;*Engase*^−/−^	**16** (18.18) [25.00]	**0**
E17.5	*Ngly1*^+/+^	**21** (28.77) [25.00]	**0**
	*Ngly1*^−/+^	**38** (52.05) [50.00]	**1**
	*Ngly1*^−/−^	**14** (19.18) [25.00]	**7**
E18.5	*Ngly1*^+/+^	**23** (31.08) [25.00]	**0**
	*Ngly1*^−/+^	**38** (51.35) [50.00]	**1**
	*Ngly1*^−/−^	**13** (17.57) [25.00]	**5**
P0	*Ngly1*^+/+^	**22**	−
	*Ngly1*^−/+^	**35**	−
	*Ngly1*^−/−^	**0**	−
2 wks	*Ngly1*^+/+^	**49**	−
	*Ngly1*^−/+^	**84**	−
	*Ngly1*^−/−^	**0**	−

E: Embryonic day, P: Postnatal day, wks: weeks, V: Viable, IV: Inviable.

(number): birth rates (%), [number]: expected (%).

### A ventricular septal defect is observed in *Ngly1*^*−/−*^ embryos

**Fig 2:**
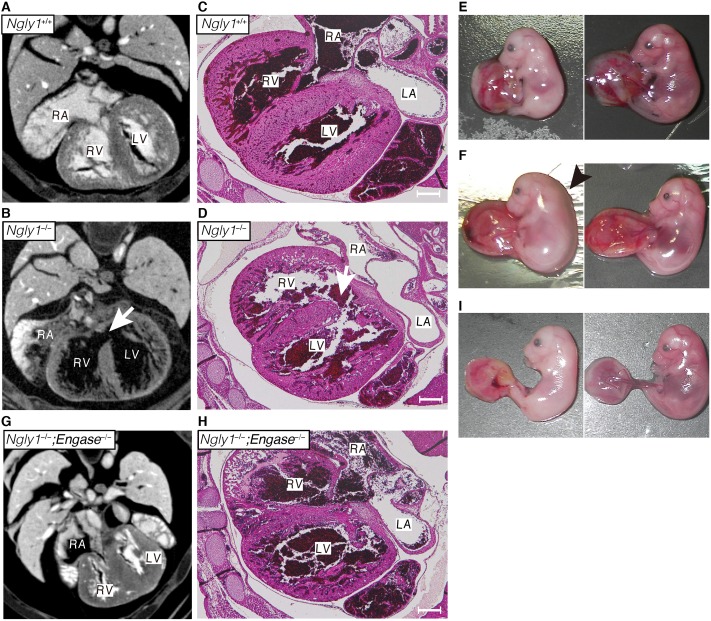
Loss of Ngly1 causes ventricular septal defects (VSD) and the additional *Engase* deletion rescues the VSD phenotypes. **(A, B, G)** Maximum intensity projection of heart μ-CT images of E16.5 embryo of wild-type (A), *Ngly1*^*−/−*^ (B), and *Ngly1*^*−/−*^*;Engase*^*−/−*^ (G). White arrows in (B) indicates VSD. **(C, D, H)** Transverse section of wild-type (C), *Ngly1*^*−/−*^ (D), and *Ngly1*^*−/−*^*;Engase*^*−/−*^ embryo (H) at E16.5 were stained with H&E. White arrows in (D) indicate VSD. Shown are representative sections (n = 3). Scale bar in (C), (D) and (H) indicate 200 μm. RA: right atrium, RV: right ventricle, LA: left atrium, LV: left ventricle. **(E)** Anemia was observed in *Ngly1*^*−/−*^ embryo at E16.5 (left panel). Right panel shows *Ngly1*^*+/+*^ embryo at E16.5 (littermate of the left panel). **(F)** Edema was observed in *Ngly1*^*−/−*^ embryo at E16.5 (left panel). Right panel shows *Ngly1*^*+/+*^ embryo at E16.5 (littermate of the left panel). Black arrowhead indicates edema. **(I)** Anemia was observed in *Ngly1*^*−/−*^*;Engase*^*−/−*^ embryo at E16.5 (left panel). Right panel shows *Ngly1*^*+/+*^*;Engase*^*−/−*^ embryo at E16.5 (littermate of the left panel). Representative images were shown.

To investigate the defects in *Ngly1*^*−/−*^ embryos in more detail, X-ray micro-computed tomography (μ-CT) analyses were carried out on *Ngly1*^*−/−*^ or *Ngly1*^*+/+*^ embryos at E16.5 (Fig 2A and 2B). As shown in Fig 2B, the *Ngly1*^*−/−*^ embryos showed a ventricular septal defect (VSD) (5 out of 5 embryos (5/5)). Histological analyses also confirmed the occurrence of a VSD in *Ngly1*^*−/−*^ embryos (3/3) (Fig 2C and 2D). VSD is one of the most frequently-observed cardiovascular phenotypes in embryonic/perinatal lethal mice [29]. We also found that some *Ngly1*^*−/−*^ embryos showed anemia (12/28 [42.86%], Fig 2E, left panel) or edema (4/28 [14.29%], Fig 2F, left panel), which were not observed in *Ngly1*^*+/+*^ embryos (0/44, Fig 2E and 2F, right panel).

### The additional deletion of the *Engase* gene partially rescues the lethality caused by the loss of *Ngly1*

In sharp contrast to the case of *Ngly1*^*−/−*^ mice, the *Engase*^*−/−*^ mice showed normal behavior/values in several tests as follows: behavior test (open field), morphology/behavioral/sensory test (RIKEN modified-SHIRPA), hematology/clinical chemistry test (hematology, urinalysis, clinical blood chemistry), pathology test (body weight), cardiovascular test (blood pressure, electrocardiogram) and neurology/psychiatry test (light/dark transition, home-cage activity, tail suspension, hot plate, tail flick) (the number of tested mice are described in Materials and Methods). According to our previous cell-based study using an ERAD model substrate [27], we hypothesized that ENGase could have the ability to function as a deglycosylating enzyme and that its deletion could rescue the defects caused by the lack of *Ngly1*. In this study, we attempted to verify the effect of the additional *Engase* deletion of *Ngly1*^*−/−*^ in mice at an individual level. To this end, we crossed *Ngly1*^*−/+*^*;Engase*^*−/+*^ or *Ngly1*^*−/+*^*;Engase*^*−/−*^ mice and found that surviving *Ngly1*^*−/−*^ mice were produced upon the crossing (Table 2). Upon further examination, all of the surviving mice were found to be *Ngly1*^*−/−*^*;Engase*^*−/−*^ double-knockout mice, strongly indicating that the additional deletion of *Engase* partially rescued the lethality caused by the defect of *Ngly1* at an individual level.

**Table 2:** Genotyping results of pups by crossing of *Ngly1*^*−/+*^*;Engase*^*−/+*^ mice or *Ngly1*^*−/+*^*;Engase*^*−/−*^ mice in C57BL/6 background.

*Ngly1*^*−/+*^*;Engase*^*−/+*^	× *Ngly1*^*−/+*^*;Engase*^*−/+*^	
**Genotype**	**Number of pups (%)**	**Expected %**
*Ngly1*^*+/+*^*;Engase*^*−/−*^	**11** (9.40)	6.25
*Ngly1*^*−/+*^*;Engase*^*−/−*^	**20** (17.09)	12.50
*Ngly1*^*−/−*^*;Engase*^*−/−*^	**3** (2.56)*	6.25
*Ngly1*^*+/+*^*;Engase*^*+/−*^	**20** (17.09)	12.50
*Ngly1*^*−/+*^*;Engase*^*+/−*^	**34** (29.06)	25.00
*Ngly1*^*−/−*^*;Engase*^*+/−*^	**0** (0.00)***	12.50
*Ngly1*^*+/+*^*;Engase*^*+/+*^	**8** (6.84)	6.25
*Ngly1*^*−/+*^*;Engase*^*+/+*^	**21** (17.95)	12.50
*Ngly1*^*−/−*^*;Engase*^*+/+*^	**0** (0.00)***	6.25
*Ngly1*^*−/+*^*;Engase*^*−/−*^	× *Ngly1*^*−/+*^*;Engase*^*−/−*^	
**Genotype**	**Number of pups (%)**	**Expected %**
*Ngly1*^*+/+*^*;Engase*^*−/−*^	**73** (28.52)	25.00
*Ngly1*^*−/+*^*;Engase*^*−/−*^	**160** (62.5)***	50.00
*Ngly1*^*−/−*^*;Engase*^*−/−*^	**23** (8.98)***	25.00

Statistic difference to expected value was examined by Chi-squared test

*p<0.05

**p<0.01

***p<0.005.

### VSD appears to be one of the critical causes of lethality in *Ngly1*^*−/−*^ mice

We next investigated the issue of whether VSD was present in *Ngly1*^*−/−*^;*Engase*^*−/−*^ embryos at E16.5. If VSD was to be critical for the lethality of *Ngly1*^*−/−*^ embryos, then this phenotype should be suppressed by the additional deletion of *Engase*. As we expected, VSD was not observed in the *Ngly1*^*−/−*^;*Engase*^*−/−*^ embryos, as evidenced by μ-CT (0/2) and histological analyses (0/2) (Fig 2G and 2H). It is therefore possible that VSD is at least one of the critical phenotypes that cause the lethality of *Ngly1*^*−/−*^ mice/embryos and the deletion of the *Engase* gene, for unknown reasons, rescues this phenotype. We also examined the gross morphology of embryos at E16.5 and found that some *Ngly1*^*−/−*^;*Engase*^*−/−*^ embryos showed anemia (5/16 [31.25%], Fig 2I, left panel) while no *Ngly1*^*+/+*^;*Engase*^*−/−*^ embryo showed this phenotype (0/20, Fig 2I, right panel). Additionally, *Ngly1*^*−/−*^;*Engase*^*−/−*^ embryos did not show edema (0/16). Unexpectedly, there is no significant difference of the appearance ratio between *Ngly1*^*−/−*^ embryos and *Ngly1*^*−/−*^;*Engase*^*−/−*^ embryos at E16.5 (Table 1). These results suggest that *Engase* deletion resulted in the rescue of not all but several phenotypes caused by *Ngly1* deletion such as VSD and edema. These results also indicated that multiple pathways appear to be involved in the pathophysiology of *Ngly1*^*−/−*^ mice.

### *Ngly1*^*−/−*^*;Engase*^*−/−*^ mice can survive but they still show several progressive defects similar to the symptoms of human subjects with an *NGLY1*-deficiency

**Fig 3:**
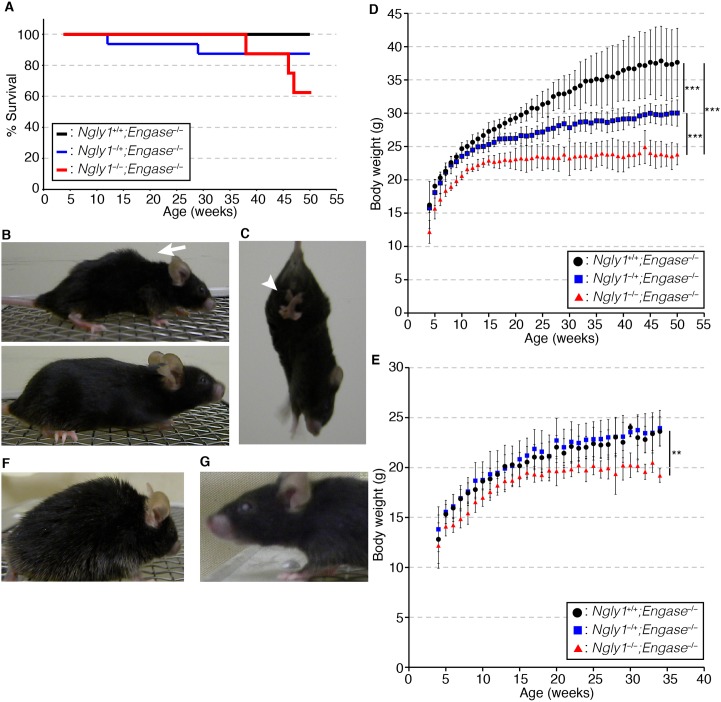
*Ngly1*^*−/−*^*;Engase*^*−/−*^ mice survive but still show several defects **(A)** Survival curve of *Ngly1*^*−/−*^*;Engase*^*−/−*^ male mice (n = 8), *Ngly1*^*−/+*^*;Engase*^*−/−*^ male mice (n = 16), and *Ngly1*^*+/+*^*;Engase*^*−/−*^ male mice (n = 7) in the C57BL/6 background. **(B)** Macroscopic comparison of 10 months-old *Ngly1*^*−/−*^*;Engase*^*−/−*^ mice (upper panel) and *Ngly1*^*+/+*^*;Engase*^*−/−*^ mice (lower panel). White arrow indicates a bent spine. **(C)** Hind-limb clasping of 5 months-old *Ngly1*^*−/−*^*;Engase*^*−/−*^ mice. White arrowhead indicates clasped hind-limb. **(D, E)** Change of body weight of *Ngly1*^*−/−*^*;Engase*^*−/−*^ mice, *Ngly1*^*−/+*^*;Engase*^*−/−*^ mice, and *Ngly1*^*+/+*^*;Engase*^*−/−*^ mice in the C57BL/6 background. (D) shows the results of male mice (n = 3~5) and (E) shows the results of female mice (n = 3~5). For statistical analysis, Student’s t-test was used. ***:p<0.001, **:p<0.01 **(F)** *Ngly1*^*−/−*^*;Engase*^*−/−*^ mice developed coarse fur (n = 12). **(G)** Female *Ngly1*^*−/−*^*;Engase*^*−/−*^ mice developed eye opacity (n = 8). Shown are representative appearances.

We next analyzed the phenotypes of the viable *Ngly1*^*−/−*^*;Engase*^*−/−*^ mice in C57BL/6 background. When the mice were tested for viability, an apparent decrease in the viability of the *Ngly1*^*−/−*^*;Engase*^*−/−*^ mice was observed after 45 weeks (about 10 months), suggesting that they show age-related defects (Fig 3A). We then attempted to characterize the major defects that were observed in *Ngly1*^*−/−*^*;Engase*^*−/−*^ mice. When they were young (about 4~5 weeks of age), the differences between the *Ngly1*^*−/−*^*;Engase*^*−/−*^ mice and their littermates were negligible. However, when they became older (about 6~7 months of age), the *Ngly1*^*−/−*^*;Engase*^*−/−*^ mice showed several defects/abnormal behavior, such as bent spines (Fig 3B, upper panel, 15/15), trembling (S1 and S2 Movies, 15/15), hind-limb clasping (Fig 3C, S3 Movie, 15/15), and front-limb shaking (S3 Movie, 15/15). Moreover, the *Ngly1*^*−/−*^*;Engase*^*−/−*^ mice showed a significant reduction in body weight compared to their littermates (Fig 3D and 3E). We currently have no explanation for why only male *Ngly1*^*−/+*^*;Engase*^*−/−*^ mice show a slow weight gain relative to *Ngly1*^*+/+*^*;Engase*^*−/−*^ mice, but it may, in part, be due to the fact that male mice are larger than female mice and therefore make a change in weight gain could be detected more easily. We observed no other haploinsufficient effects in male/female *Ngly1*^*−/+*^*;Engase*^*−/−*^ mice.

*Ngly1*^*−/−*^*;Engase*^*−/−*^ mice also had coarse fur (12/15 [80%]) which may be due to either intrinsic skin problems or, alternatively, impaired grooming behavior (Fig 3F). Female *Ngly1*^*−/−*^*;Engase*^*−/−*^ mice exhibited eye opacity much more frequently than their littermates after 20~30 weeks of age (8/10 [80%], Fig 3G). Those results clearly indicate that, while the deletion of the *Engase* gene results in suppressing the critical phenotypes, including the VSD caused by deletion of the *Ngly1* gene, not all of the defects were fully rescued.

### A change in the genetic background of C57BL/6 mice can partially rescue the embryonic lethality caused by the loss of Ngly1

**Fig 4:**
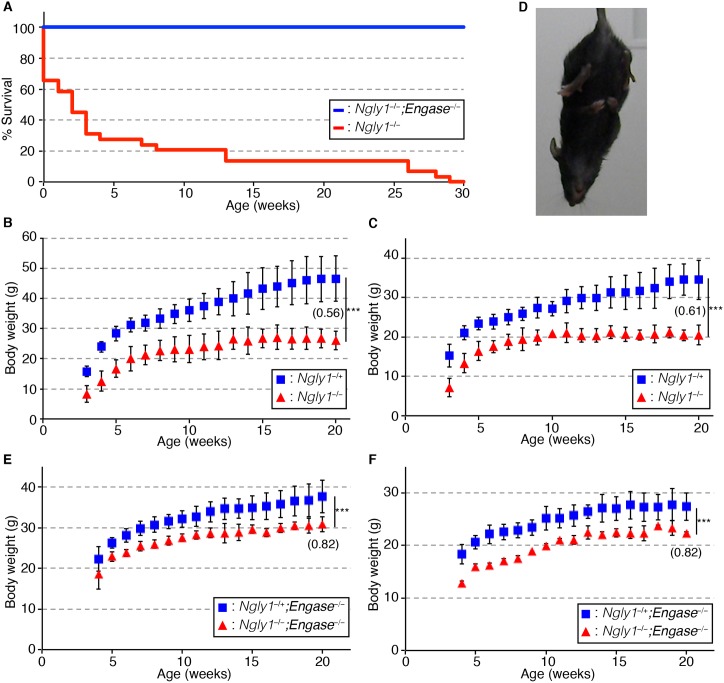
*Engase*-deletion improves the % survival and partially rescues the body weight loss of viable *Ngly1*^*−/−*^ mice. **(A)** Survival curve for *Ngly1*^*−/−*^*;Engase*^*−/−*^ mice (n = 9) and *Ngly1*^*−/−*^ mice (n = 29) in the C57BL/6 and ICR mixed background. **(B, C)** Change of body weight of *Ngly1*^*−/+*^ mice and *Ngly1*^*−/−*^ mice in C57BL/6 and ICR mixed background. (B) shows the results of male mice (*Ngly1*^*−/+*^:n = 3~4, *Ngly1*^*−/−*^:n = 2~5) and (C) shows the results of female mice (*Ngly1*^*−/+*^:n = 5~11, *Ngly1*^*−/−*^:n = 2~5). **(D)** Hind-limb clasping of 4 weeks-old *Ngly1*^*−/−*^ mice in the C57BL/6 and ICR mixed background. **(E, F)** Change in body weight of *Ngly1*^*−/+*^*;Engase*^*−/−*^ mice and *Ngly1*^*−/−*^*;Engase*^*−/−*^ mice in the C57BL/6 and ICR mixed background. (E) shows the results of male mice (*Ngly1*^*−/+*^*;Engase*^*−/−*^:n = 4~8, *Ngly1*^*−/−*^*;Engase*^*−/−*^:n = 2~4) and (F) shows the results of female mice (*Ngly1*^*−/+*^*;Engase*^*−/−*^:n = 7~11, *Ngly1*^*−/−*^*;Engase*^*−/−*^:n = 2~3). For statistical analysis, Student’s t-test was used. ***:p<0.001. The number in panel B, C, E, and F indicates the relative ratio of weight of *Ngly1*^*−/−*^ mice to *Ngly1*^*−/+*^ mice at 20 weeks of age.

It was previously shown that a change in the genetic background of *Fut8*^*−/−*^ mice from an inbred C57BL/6 strain to an outbred ICR strain, that maintain a certain genetic variation within the colonies, postnatal lethality was rescued [30]. It is also noteworthy that, when we examined the symptoms of *NGLY1*-deficiency patients, there were a wide spectrum of symptoms and a genotype-phenotype correlation was not so obvious [23–25]. These results imply that the genetic background may influence the severity of the phenotypes caused by the defects of *Ngly1*. With that in mind, we crossed *Ngly1*^−/+^ mice in a C57BL/6 background with ICR mice. Among the F1 mice that were produced, the *Ngly1*^−/+^ mice were further crossed to produce F2 mice. As shown in Table 3, we found that viable F2 *Ngly1*^*−/−*^ mice were obtained from these crosses. The phenotypes of the *Ngly1*^*−/−*^ mice in the C57BL/6 and ICR mixed background were found to be much more severe compared with the *Ngly1*^*−/−*^*;Engase*^*−/−*^ mice in the C57BL/6 background, and about 70% of them died within 3 weeks (Fig 4A). The *Ngly1*^*−/−*^ mice in the C57BL/6 and ICR mixed background also showed a significant reduction in body weight (Fig 4B and 4C) and hind-limb clasping (Fig 4D, 9/9), which were also observed in aged *Ngly1*^*−/−*^*;Engase*^*−/−*^ mice in the C57BL/6 background (Fig 3C–3E). It should be noted that the deletion of the *Engase* gene resulted in a slight reduction in the body weight in mice of the C57BL/6 and ICR mixed background (ex. compare Fig 3B, 3C, 3D and 3E for *Ngly1*^*−/+*^ mice). This observation suggests that the loss of the *Engase* gene affects weight gain in mice. We suspect, however, that this fluctuation in the data could be simply due to the diverse genetic background of the mice that were analyzed, especially since ICR mice are, in general, much larger than C57BL/6 mice. Consistent with this assumption, pathological tests at the Japan mice clinic showed that the average weight of 26-week old *Engase*^*−/−*^ mice (average 26.64/26.61 g for male/female mice (n = 5)) was not affected when compared with that of wild-type mice (26.79/26.64 g for male/female mice (n = 1)) with the C57BL/6 background. However, we currently cannot exclude the possibility that the loss of the *Engase* gene may become problematic when *Ngly1* levels are compromised.

**Table 3:** Genotyping results of pups by the crossing of *Ngly1*^*−/+*^ mice or *Ngly1*^*−/+*^*;Engase*^*−/−*^ mice in C57BL/6 and ICR mixed background.

*Ngly1*^*−/+*^ × *Ngly1*^*−/+*^		
**Genotype**	**Number of pups (%)**	**Expected %**
*Ngly1*^*+/+*^	60 (30.46)	25.00
*Ngly1*^*−/+*^	106 (53.81)	50.00
*Ngly1*^*−/−*^	31 (15.73)***	25.00
*Ngly1*^*−/+*^*;Engase*^*−/−*^	×*Ngly1*^*−/+*^*;Engase*^*−/−*^	
**Genotype**	**Number of pups (%)**	**Expected %**
*Ngly1*^*+/+*^*;Engase*^*−/−*^	20 (27.40)	25.00
*Ngly1*^*−/+*^*;Engase*^*−/−*^	44 (60.27)	50.00
*Ngly1*^*−/−*^*;Engase*^*−/−*^	9 (12.33)*	25.00

Statistic difference to expected value was examined by Chi-squared test

*p<0.05

**p<0.01

***p<0.005.

In the case of the *Ngly1*^*−/−*^ mice in the C57BL/6 and ICR mixed background, however, they began to show these abnormalities when they were still young (4 weeks old). Therefore, the phenotype of the *Ngly1*^*−/−*^ mice in the C57BL/6 and ICR mixed background turned out to be much more severe than that of *Ngly1*^*−/−*^*;Engase*^*−/−*^ mice in the C57BL/6 background, even when they partially evaded embryonic lethality. These results also indicate the suppressive effects of the deletion of *Engase* for phenotypes of *Ngly1*^*−/−*^ mice, since mice with the C57BL/6 and ICR mixed background are generally thought to be more tolerant to genetic mutations when compared with the inbred C57BL/6 background.

To determine if the partial suppression of lethality in the ICR and C57BL/6 mixed background may have been caused by the low ENGase activity in ICR mice, we carried out ENGase enzyme assays using PA-labeled sugar derivatives on liver tissue obtained from ICR and C57BL/6 mice. The findings indicated that the samples from the ICR and C57BL/6 mice had equivalent ENGase activity (0.39±0.11 pmol/min/mg protein for C57BL/6 and 0.42±0.07pmol/min/mg protein for ICR), and no ENGase activity was detected in the liver of *Engase*^*−/−*^ mouse. These results indicate that the rescue of the lethality of *Ngly1*^*−/−*^ mice resulting from a change in their genetic background does not appear to be correlated with ENGase activity.

### The deletion of *Engase* can partially alleviate the defects caused by *Ngly1* deletion in viable *Ngly1*^−/−^ mice

To test the effect of *Engase* deletion in the viable *Ngly1*^*−/−*^ mice, we next generated *Ngly1*^*−/−*^*;Engase*^*−/−*^ mice in the C57BL/6 and ICR mixed background. As shown in Table 3, we were able to obtain *Ngly1*^*−/−*^*;Engase*^*−/−*^ mice in the F3 mice by crossing the F2 *Ngly1*^*−/+*^*;Engase*^*−/−*^ mice in the C57BL/6 and ICR mixed background. All of the F3 *Ngly1*^*−/−*^*;Engase*^*−/−*^ mice were alive at 30 weeks (n = 9), in sharp contrast to the case with the F2 *Ngly1*^*−/−*^ mice in the C57BL/6 and ICR mixed background, where none of them had survived at 30 weeks (n = 29) (Fig 4A). Moreover, the *Ngly1*^*−/−*^*;Engase*^*−/−*^ mice showed an attenuated body weight reduction compared to the *Ngly1*^*−/−*^ mice in the C57BL/6 and ICR mixed background (Fig 4E and 4F). These results clearly indicate that the disruption of ENGase can alleviate, at least partially, the defects caused by Ngly1 deletion in the viable mice (e.g. reduction of % survival and body weight). The phenotypes of the *Ngly1*^*−/−*^*;Engase*^*−/−*^ mice from this cross were found to be even more mild than the *Ngly1*^*−/−*^*;Engase*^*−/−*^ mice in the C57BL/6 background, in that they did not show any overt defects, other than hind-limb clasping (9/9), suggesting that the deletion of the *Engase* gene and mixing with ICR-background have an additive effect in suppressing the defect of the *Ngly1*^*−/−*^ mice.

### Identification of *N*-glycoproteins accumulated in the cytoplasm of *Ngly1*^*−/−*^*;Engase*^*−/−*^ and wild-type MEF cells

**Fig 5:**
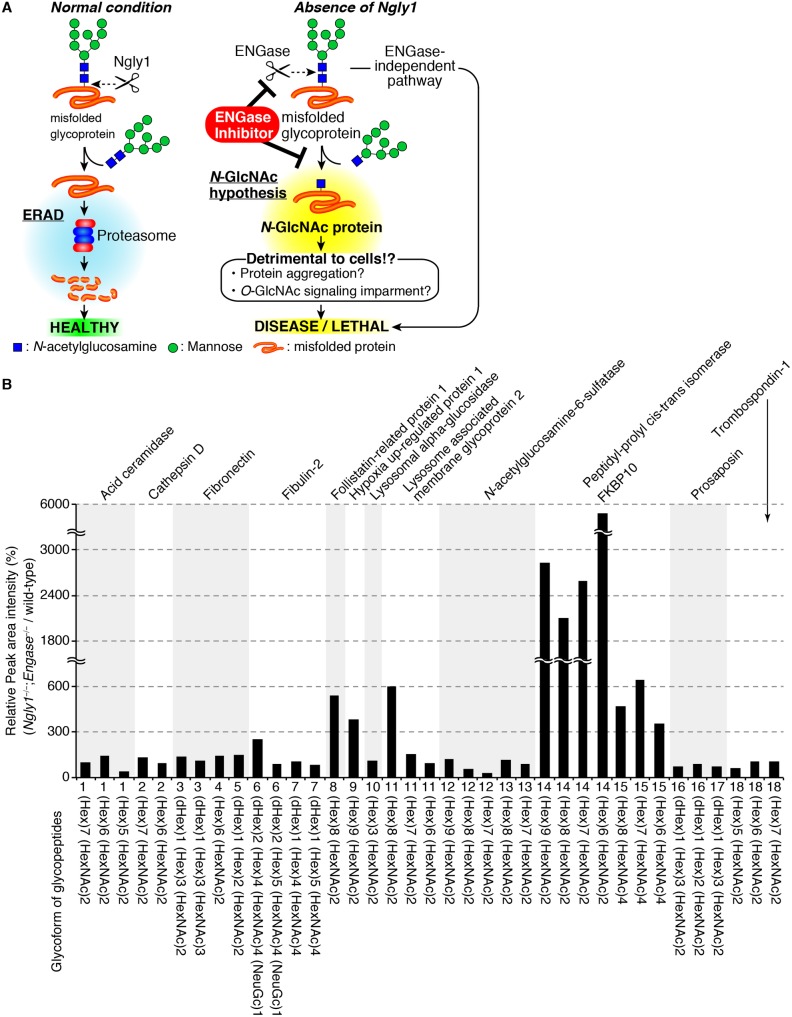
*N*-GlcNAc hypothesis and the accumulation of *N*-glycoproteins in the cytoplasm of *Ngly1*^*−/−*^*;Engase*^*−/−*^ MEF cells. **(A)** Schematic representation of an *N*-GlcNAc hypothesis and therapeutic treatment of *NGLY1*-deficiency based on ENGase inhibition. In the absence of Ngly1, ENGase acts on some portions of unfolded glycoproteins to form *N*-GlcNAc proteins. The presence of an excess of *N*-GlcNAc proteins somehow results in detrimental effects on cells/mice. (B) Relative peak area intensity (%) of glycopeptides observed in *Ngly1*^*−/−*^*;Engase*^*−/−*^ MEF cells. The peak area of each glycopeptide in wild-type MEF cells was identified as 100% and the relative ratio of the glycopeptide peak area in *Ngly1*^*−/−*^*;Engase*^*−/−*^ MEF cells were calculated. Shown are the average values for two samples that were independently prepared.

Regarding the potential mechanism for how ENGase can rescue the defects due to the loss of *Ngly1*, we previously proposed an “*N-*GlcNAc hypothesis” [27]. In this proposed process, ENGase acts on cytoplasmic misfolded glycoproteins in a stochastic fashion, especially when Ngly1 activity is compromised. As a result, *N-*GlcNAc modified proteins (proteins that are modified with one GlcNAc unit) would be generated in the cytoplasm. The formation of the excessive levels of *N-*GlcNAc proteins could somehow lead to detrimental effects (Fig 5A). We also previously showed that there appears to be higher levels of cytoplasmic *N*-glycoproteins in *Ngly1*^*−/−*^*;Engase*^*−/−*^ MEF cells compared with wild-type MEF cells, as judged by lectin blot experiments [27]. Such cytoplasmic *N*-glycoproteins may serve as precursors for endogenous *N-*GlcNAc proteins, some of which may be linked to the pathogenesis of an *NGLY1*-deficiency, in *Ngly1*^*−/−*^ cells.

To identify specific *N-*glycoproteins that accumulate in *Ngly1*^*−/−*^*;Engase*^*−/−*^ MEF cells, a glycoproteomics analysis was carried out on the cytoplasm of *Ngly1*^*−/−*^*;Engase*^*−/−*^ MEF cells. To minimize contamination from the membrane fraction, we utilized a mild digitonin-treatment to recover the cytoplasmic fraction [31]. The cytoplasmic fractions thus prepared were subjected to a glycoproteomics analysis. After trypsinization, the *N-*glycopeptides were concentrated by acetone precipitation [32], and the *N-*glycans were released by PNGase F digestion, followed by the detection of deglycosylated peptide in which *N-*glycosylated asparagines are converted into aspartic acids. Among the 79 potential *N-*glycopeptides that were detected (S2 and S3 Tables), 13 *N*-glycopeptides were detected as deamidated peptides, even in the absence of PNGase F-treatment (S3 Table), indicating that those 13 glycopeptides are formed by deamidation of the asparagine residues in a non-enzymatic fashion. The presence of *N-*glycans on the remaining 66 peptides (S2 Table) was further confirmed by the detection of signals for *N-*GlcNAc peptide ions on LC/MS/MS analysis of PNGase F-untreated sample [32]. Through this analysis, 18 *N-*glycopeptides derived from 12 glycoproteins were unequivocally identified in the *Ngly1*^*−/−*^*;Engase*^*−/−*^ cytoplasm (Table 4, S1 Fig for product ion spectra). A comparison of the MS peak areas of these glycopeptides between *Ngly1*^*−/−*^*;Engase*^*−/−*^ and wild-type revealed that larger signals were detected for several *N-*glycopeptides in the *Ngly1*^*−/−*^*;Engase*^*−/−*^ cytoplasm samples (Table 4, Fig 5B), while our analysis is by no means quantitative and the difference in the amount of *N*-glycoproteins in the two different cells may also be due to the differential recovery of the *N-*glycoproteins during the concentration of the *N*-glycoproteins. Nevertheless, our data clearly indicates that, indeed, the cytoplasm did contain *N-*glycoproteins, which may become potential substrates for Ngly1/ENGase in the cytoplasm and therefore can serve as precursors for *N-*GlcNAc proteins.

**Table 4:** Detected *N*-glycopeptides in the cytoplasm of *Ngly1*^*−/−*^*;Engase*^*−/−*^ MEF cells.

Protein name	#	Peptides sequences*
Acid ceramidase	1	SVLE *N* TTSYEEAK
Cathepsin D	2	YYHGELSYL *N* VTR
Fibronectin	3	*N* YTDCTSEGR
	4	HEEGHML *N* CTCFGQGR
	5	LDAPTNLQFV *N* ETDR
Fibulin-2	6	EGETCGAED *N* DTCGVSLYK
	7	DLDECALGTH *N* CSEAETCHNIQGSFR
Follistatin-related protein 1	8	GS *N* YSEILDK
Hypoxia up-regulated protein 1	9	VFGSQ *N* LTTVK
Lysosomal alpha-glucosidase	10	QVVE *N* MTR
Lysosomal-associated membrane glycoprotein 2	11	VQPF *N* VTK
*N*-acetylglucosamine-6-sulfatase	12	GPGIKP *N* QTSK
	13	TPMT *N* SSIR
Peptidyl-prolyl cis-trans isomerase FKBP10	14	TLSRPPENC *N* ETSK
	15	YHY *N* GTFEDGK
Prosaposin	16	T *N* SSFIQGFVDHVK
	17	D *N* ATQEEILHYLEK
Thrombospondin-1	18	VV *N* STTGPGEHLR

*N in the peptide sequences indicates *N*-glycosylation site.

## Discussion

In this study, the functional significance of the Ngly1 gene in mice were characterized. The results are consistent with the recent reports on *NGLY1-*deficient human subjects bearing mutations in the *NGLY1* allele [22–26]. Surprisingly, the lethality of the *Ngly1*^*−/−*^ mice was partially suppressed by the additional knockout of the *Engase* gene. This result is consistent with findings reported in an earlier study [27], which showed that the disruption of ERAD in *Ngly1*-KO cells was restored by the additional deletion of *Engase*, at an individual level. Through analyses of various mutants for *Ngly1* gene orthologues, an enzyme-independent function of the Ngly1-orthologue was proposed for some organisms [15, 18, 33]. The results reported herein clearly suggest, however, that the embryonic lethal phenotype of *Ngly1*^*−/−*^ mice is mainly related to its deglycosylation-dependent function, given the fact that the lethality was partially suppressed by the additional deletion of *Engase*, a gene encoding another cytoplasmic deglycosylating enzyme.

Regarding the ventricular septum defect observed for *Ngly1*^*−/−*^ embryos, it should be noted that no significant heart problems have been reported for human patients [22–26], suggesting that the functional importance of *Ngly1* for the heart may be limited to its embryonic developmental stage. In this connection, it is also noteworthy that one of the patients was diagnosed as “small drop out in interventricular septum” by a fetal echo analysis (personal communication from Mr. Andreas Thermann).

While the lethality of the *Ngly1*^*−/−*^ mice was partially rescued by the additional deletion of *Engase* or by changing the genetic background, recognizable phenotypes were still observed in the *Ngly1*^*−/−*^*;Engase*^*−/−*^ mice, as well as the *Ngly1*^*−/−*^ mice in the C57BL/6 and ICR mixed background. It should be noted here that most of the phenotypes observed for those mice are reminiscent of the symptoms observed for an *NGLY1-*deficiency in human subjects (S4 Table). Since the *Ngly1*^*−/−*^ mice are embryonic lethal in the C57BL/6 background, the *Ngly1*^*−/−*^*;Engase*^*−/−*^ mice as well as the *Ngly1*^*−/−*^ mice in the C57BL/6 and ICR mixed background could serve as viable model animals for studying the functions of Ngly1, which could be of great value, especially for evaluating the efficacy of potential drugs for the treatment of *NGLY1*-deficiency subjects in the future.

With respect to the potential mechanism responsible for the detrimental effect of ENGase in *Ngly1*^*−/−*^ mice, we previously proposed an *N*-GlcNAc hypothesis as described above [27]. Such *N*-GlcNAc proteins were recently reported to be present in murine synaptosome proteins [34]. In addition, in plants, at least one of the *N-*GlcNAc proteins was shown to be generated by the action of the cytoplasmic ENGase [35]. There was also a report suggesting that the proximal *N*-GlcNAc on *N*-glycosylation sites has a critical role in stabilizing carrier proteins [36]. We therefore hypothesize that ENGase might cleave glycans on misfolded glycoproteins in the cytoplasm to generate *N*-GlcNAc proteins, and that the presence of an excess of these *N*-GlcNAc proteins somehow contributes to the detrimental effects in mice, such as *N*-GlcNAc proteins forming aggregates, or impairment of cytoplasmic *O-*GlcNAc signaling (*N*-GlcNAc hypothesis) (Fig 5A). Indeed, we found that a model ERAD substrate was converted into an *N*-GlcNAc-modified protein in *Ngly1*^*−/−*^ MEF cells [27]. In this connection, it should be noted that a GalNAc-GalNAc (GalNAc: *N*-acetylgalactosamine) interaction was recently reported to occur in the binding of mucins with Tn (GalNAc α1-Ser/Thr) or sialyl Tn cancer antigens [37]. It would be interesting to see if similar interactions, at least in part, contribute to the aggregation of *N*-GlcNAc proteins. On the other hand, we have not been able to detect *Ngly1*-deficient specific change in the *O*-GlcNAc modification among MEF cells by western blotting (S2 Fig). However, the possibility that changes in specific *O*-GlcNAc proteins had occurred cannot be excluded. Clearly, a more comprehensive analysis will be required. Moreover, *Ngly1*-deficient specific protein aggregation was not observed by histological analyses (e.g. Congo red staining, PAS staining) of tissue sections from mice embryos. Further studies will be needed to verify our *N*-GlcNAc hypothesis and efforts are currently underway to identify endogenous *N*-GlcNAc proteins in MEF cells derived from various genotypes (wild-type, *Ngly1*^*−/−*^, *Engase*^*−/−*^ and *Ngly1*^*−/−*^*;Engase*^*−/−*^). It should be noted that *Ngly1-*KO does not lead to a general ERAD-defect [8, 9, 38]. These observations strongly indicate that the pathogenesis of *Ngly1*^*−/−*^ mice cannot be attributed to general ER stress, but rather specific substrates likely appear to be involved.

We previously reported that larger amounts of *N-*glycoproteins appeared to be accumulated in the cytoplasm of *Ngly1*^*−/−*^*;Engase*^*−/−*^ MEF cells compared with those of wild-type, *Ngly1*^*−/−*^ or *Engase*^*−/−*^ MEF cells [27]. To extend our analysis, in this study we identified several *N*-glycoproteins that had accumulated in the cytoplasm of *Ngly1*^*−/−*^*;Engase*^*−/−*^ cells. Such glycoproteins can serve as precursors for endogenous *N-*GlcNAc proteins that are potentially formed in *Ngly1*^*−/−*^ cells. While we used sequential detergent extraction for the cytoplasmic fraction to minimize contamination of other organelles, traces of lysosomal components could still be detected in the cytoplasmic fraction (S3 Fig). It is therefore possible that some glycoproteins of lysosomal origin may be present due to contamination by lysosomes in the cytoplasmic fraction.

In any event, the phenotypes observed for *Ngly1*^*−/−*^ and *Ngly1*^*−/−*^*;Engase*^*−/−*^ mice were found to be very analogous to the symptoms observed in patients with an *NGLY1*-deficiency (S4 Table), which make those mice valuable animal models for this genetic disorder. It should also be noted that the degree of suppression by *Egnase-*deletion are distinct among phenotypes (ex Fig 2). These observations clearly suggest that multiple pathways, *i*.*e*. ENGase-dependent and–independent pathways, are involved in the pathogenesis of *Ngly1-*KO mice or *NGLY1-*deficiency subjects.

Since many of the *Ngly1*^*−/−*^*;Engase*^*−/−*^ mice in both the C57BL/6 background and the C57BL/6 and ICR mixed background survive for more than 1 year after birth (one lived for more than 2 years), this either suggests that (1) once they escape the developmental defect during embryogenesis, the *Ngly1*^*−/−*^ mice could have a relatively normal life span or (2) the effect of suppression by *Engase*-deletion is quite effective for adult mice. However, the *Ngly1*^*−/−*^ mice in the C57BL/6 and ICR mixed background showed quite severe phenotypes, favoring the latter possibility. Moreover, the additional deletion of *Engase* in the C57BL/6 and ICR background also resulted in a significant improvement of the phenotypes (% survival, body weight gain), further supporting the conclusion that a defect in the *Engase-*gene has strong positive effects for viable mice. In this connection, it should be noted that, while the birth rate of *Ngly1*^*−/−*^*;Engase*^*−/−*^ mice from *Ngly1*^*−/+*^*;Engase*^*−/−*^ cross was found to be better in the C57BL/6 and ICR mixed background (12.33%) than the C57BL/6 homogeneous background (8.98%) (Tables 2 and 3), it was still significantly lower than the expected rate (25%). These results clearly suggest that the deletion of *Engase* is not sufficient to fully suppress the *Ngly1* defect during embryogenesis. Further studies will be required to understand the molecular details on the functional importance of Ngly1 in embryogenesis.

Given the dramatic rescue of the mice phenotypes by the additional *Engase* deletion, one could easily envision that an *in vivo* inhibitor of cytoplasmic ENGase would be a promising therapeutic approach for the treatment of an *NGLY1*-deficiency in humans (Fig 5A). It has previously been shown that the thiazoline modified oligosaccharide functions as an effective inhibitor for the cytoplasmic ENGase [39], and it may be possible to design cell permeable inhibitors for ENGase derived from this compound.

## Materials and methods

### Ethics statement

All animal experiments were approved by the institutional committee of RIKEN (approved number is: H28-2-003(2)). All mice in this study were euthanized by cervical dislocation.

### Mice

We generated *Ngly1-* or *Engase-*deficient mice as described previously [27]. Mice were housed in a facility with access to food and water and were maintained under 12-hour light/12-hour dark cycle.

### *Engase*^*−/−*^ mice analyses

*Engase*^*−/−*^ mice analyses were performed at Japan Mouse Clinic (http://ja.brc.riken.jp/lab/jmc/mouse_clinic/en/index.html). For open field test, 18 *Engase*^+/+^ mice, 21 *Engase*^−/+^ mice and 20 *Engase*^−/−^ mice were used. For RIKEN modified-SHIRPA, hematology, urinalysis, clinical blood chemistry, pathology, blood pressure, and electrocardiogram tests, 11 *Engase*^+/+^ mice, 14 *Engase*^−/+^ mice and 14 *Engase*^−/−^ mice were used. For light/dark transition, home-cage activity, tail suspension, hot plate, and tail flick tests, 7 *Engase*^+/+^ mice, 7 *Engase*^−/+^ mice and 6 *Engase*^−/−^ mice were used.

### Cell cultures

MEF cells were isolated from E13.5 or E14.5 embryos by trypsinization [40]. The isolated MEF cells were cultivated in DMEM (4.5 g/L glucose, 10% FBS, 100 unit/mL penicillin and 100 μg/mL streptomycin) at 37°C in a 5% CO_2_ atmosphere.

### Genotyping

Genotyping to confirm the targeted disruption of Ngly1 and ENGase was performed by PCR analysis using genomic DNA extracted from the tail or amnion and 8 sets of primers, as shown in S1 Table. PCR products were separated by 2(w/v)% agarose gel electrophoresis, and the band size was analyzed. Primer set 1–2, 3–4, 5–6, 7–8 can detect *Ngly1*^*+/+*^, *Ngly1*^*−/−*^, *Engase*^*+/+*^, *Engase*^*−/−*^ allele respectively. PCR product size (band size) of each primer sets are shown in S1 Table.

### Histology

Embryos were fixed in Bouin’s solution. Fixed embryos were washed with 70% ethanol until the yellow color of picric acid was no longer visible. The washed embryos were dehydrated through a series of ethanol solutions (70%, 80%, 90%, 95%, 99% and 100%), cleared with xylene, and embedded in paraffin. Samples were sectioned at 5 μm and stained with hematoxylin and eosin (HE staining) using the general protocol.

### Micro-CT analysis

The collected embryos were fixed with a 10% neutral buffered formalin solution (SIGMA). Fixed embryos were soaked in contrast agent, a 1:3 mixture of Lugol solution and deionized distilled water and then analyzed by μ-CT. μ-CT analyses were performed as previously reported [41]. Briefly, mouse embryos were scanned using a SCANXMATE-E090 scanner (Comscantechno) at a tube voltage peak of 40 kVp and a tube current of 100 μA. Samples were rotated 360° in steps of 0.24°, generating 1500 projection images. The μ-CT data were reconstructed at an isotropic resolution of 10.5 μm. Three-dimentional, tomographic images were obtained using the OsiriX software (www.osirix-viewer.com).

### Purification of (His)_6_-tagged N-terminal mouse Ngly1 (1–181) and raising an antibody against Ngly1

An anti-mouse Ngly1 antibody was raised against the *E*. *coli* expressed N-terminus fragment of Ngly1 using pET-mPng1N [42], which express the (His)_6_-tagged N-terminal fragment of Ngly1 (aa1-181). In brief, to a 500 mL BL21(DE3)pLysS suspension of growing-phase cells carrying pET-mPng1N, was added isopropyl-β-thiogalactoside (final conc. 1 mM) at 37°C for 3 hours. The cells were then collected, suspended in 25 mL of lysis buffer (50 mM Tris-HCl buffer, pH 8.0, with 1 mM Pefabloc (Roche) and 0.15 M NaCl) with 200 μg/mL lysosyme and incubated on ice for 30 min. Cells were lysed by sonication, and a crude cell extract was obtained by removing the debris. A 12 ml portion of the protein extract (equivalent to 240 ml *E*. *coli* culture) was applied to a Profinia protein purification system with a Bio-Scale Mini Ni-IMAC Profinity column (Bio-Rad) according to the manufacturer’s protocol. The purified (His)_6_-tagged protein thus obtained (2.5 mL) was desalted by passage through a PD-10 column (GE), and the purified protein fraction thus obtained (~4 mg/mL) was used as an immunogen. The antiserum raised against the purified Ngly1 (aa1-181), as well as the affinity purified IgG fraction using the antigen-conjugated resin, was prepared by Biogate Co., Ltd (Gifu, Japan).

### Preparation of cytoplasmic fraction from MEF cells

Cultured MEF cells were washed with PBS 3 times. The following steps were carried out either on ice or at 4°C. Cells collected were suspended with 10 mM Tris-HCl buffer (pH7.5) containing 1mM ethylenediaminetetraacetic acid (EDTA), 250 mM sucrose, 1 mM dithiothreitol (DTT), 1 mM AEBSF (Pefabloc SC, Roche) and various protease inhibitors (1 × complete protease inhibitor cocktail (Roche)) at a density of 5 × 10^7^ cell/mL, and were homogenized using a Potter-Elvehjem homogenizer. The extracts thus obtained were cleared at 15,000 rpm for 10 min at 4°C and the supernatant was transferred to a new tube. The sample was ultracentrifuged at 100,000×*g* for 1 h at 4°C, and the supernatant was collected and used as the cytoplasmic fraction.

### Subcellular fractionation through sequential detergent extraction

2 × 10^6^ MEF cells were seeded on a 150 cm^2^ flask 48 h before extraction. Cultured MEF cells were washed 3 times with 10 mL of PBS. The flask was gently coated with 1 mL of permeabilization buffer (25 mM Hepes (pH 7.2), 110 mM potassium acetate, 2.5 mM magnesium acetate, 1 mM ethyleneglycoltetraacetic acid (EGTA), 1 mM DTT, 1 mM phenylmethylsulfonyl fluoride (PMSF), 0.015(wt/vol)%, digitonin) and slowly rocked at 4°C for 5 min. The buffer was collected and centrifuged at 7,500×*g* for 10 min at 4°C. The supernatant was transferred to a new tube and used as cytoplasmic fraction for glycoproteomics analyses. The flask was then washed with 5 mL of wash buffer (25 mM Hepes (pH 7.2), 110 mM potassium acetate, 2.5 mM magnesium acetate, 1 mM EGTA, 1 mM DTT, 1 mM PMSF, 0.004(wt/vol)%, digitonin). The flask was coated 1 mL of lysis buffer (25 mM Hepes (pH 7.2), 400 mM potassium acetate, 15 mM magnesium acetate, 1 mM DTT, 1 mM PMSF, 1(wt/vol)% NP-40, 0.5(wt/vol)% sodium deoxycholate) and rocked at 4 for 5 min. The buffer was collected and centrifuged at 7,500×*g* for 10 min at 4°C. The supernatant was transferred to a new tube and used as membrane fraction.

### Sample preparation for glycoproteomics analysis

The sample preparation were performed as previously described [32]. Briefly, following the addition of 4 μL of 1 M DTT, the detergent extracted cytoplasmic fractions (100 μL, 60 μg) were incubated at 65°C for 30 min. 5.6 μL of 1 M iodoacetoamide was added and the mixtures were incubated at room temperature for 40 min in the dark. The reaction was stopped by adding 2.4 μL of 1 M DTT, and then desalted by acetone precipitation prior to tryptic digestion. The precipitated samples were dissolved in 30 μL of 50 mM Tris-HCl (pH 8.5) containing 0.1(w/v)% sodium deoxycholate. Modified trypsin (3 μg) (Promega) was added to the samples, and the mixtures were incubated at 37°C for 16 h. The peptide mixtures were diluted with Milli-Q water to a final volume of 50 μL. A five-fold volume of ice cold acetone was then added to the peptides and the glycopeptide mixtures were incubated at −25°C for at least 16 h to precipitate the glycopeptides (glycopeptides enrichment). The precipitated glycopeptide fractions were collected by centrifugation at 12,000×*g* for 10 min at 20°C, and the precipitated glycopeptides fractions were dissolved in Milli-Q water. A part of the glycopeptides fractions were desalted using a Pierce C-18 Spin Column (Thermo Scientific) following the manufacturer’s instructions and used for glycopeptide analyses. A second portion of the glycopeptide fraction was dissolved in 50 μL of 50 mM phosphate buffer (pH 7.2) containing 10 mM EDTA and used for analyses of deaminated peptides. To release the *N*-glycans, the sample was incubated with 1 U of PNGase F with 50% glycerol at 37°C overnight. After drying with a SpeedVac concentrator, the sample was desalted as described above. PNGase F treated samples were used for analyses of deamidated peptides to estimate the *N*-glycosylation sites on peptides.

### Liquid chromatography/mass spectrometry (LC/MS) analyses

The samples prepared were dissolved in 20 μL of 0.1% formic acid, and a 1 μL aliquot of the sample was injected into an EASY-nLC 1000 (Thermo Scientific) with an Acclaim PepMap 100 trapping column (75 μm×2 cm, nanoViper; Thermo Scientific) and a Nano HPLC Capillary Column (75 μm×120 mm, 3 μm, C18; Nikkyo Technos). The mobile phases were 0.1% formic acid (A buffer) and 0.1% formic acid in acetonitrile (B buffer). The glycopeptides were eluted at a flow rate of 0.3 μL/min with a linear gradient from 0 to 45% B over 55 min. Mass spectra were acquired on a Q Exactive mass spectrometer (Thermo Scientific) equipped with Nanospray Flex Ion Source (Thermo Scientific). The electrospray voltage was 2.0 kV, and the resolution was 70,000. Full mass scans were performed at a range of *m/z* 700–2,000, and product ion spectra were acquired data-dependently in the positive ion mode. The deglycosylated peptides were analyzed in a similar manner with the exception of full mass scans at *m/z* 350–2000.

### Identification of glycopeptides

The deglycosylated (PNGase F treated) peptides were identified by a database search analysis using the SEQUEST search engine (Thermo Scientific) with applying the following parameters: a specified trypsin enzymatic cleavage with 2 possible missed cleavages, precursor mass tolerance of 6 ppm, fragment mass tolerance of 0.02 Da, static modification of cysteine (carbamidomethylation), and dynamic modification of methionine (oxidation) and asparagine (deamidation). All of the identified peptides were then filtered at a false discovery rate (FDR) threshold of 1%. The amino acid sequences of glycopeptides were identified by matching between the product ion of peptide carrying a single *N*-acetylglucosamine residue and the identified deglycosylated peptide.

### Western blotting

Cytoplasmic fractions of MEF cells derived from *Ngly1*^*−/−*^, or wild type mice were separated by sodium dodecylsulfate (SDS)-polyacrylamide gel electrophoresis (PAGE) and electroblotted onto polyvinylidene fluoride (PVDF) membrane. The membrane was blocked for 1 h with 4(w/v)% skim milk in PBS and incubated with anti-mouse Ngly1 antibody in PBS supplemented with 0.05% (wt/vol) Tween20 (PBST) (1:3000) at 4°C for 16 h, followed by HRP goat anti-rabbit IgG (1:3000, GE Healthcare) in PBST at room temperature for 2 h. The membranes were monitored by an Immobilon Western Chemiluminescent HRP Substrate (Millipore). The chemiluminescence was visualized using a LAS-3000 Imager (Fujifilm).

### Preparation of cytoplasm from mice tissues

Livers obtained from 7-week-old female mice of ICR background (n = 3), and C57BL/6 background (n = 3), as well as a 16-week-old female C57BL/6 mouse of *Engase*^*−/−*^ (n = 1) were suspended in 2 volumes of cytoplasm extraction buffer containing 10 mM Hepes-NaOH (pH7.4), 250 mM sucrose, 1 mM DTT, 1 mM ethylenediaminetetraacetic acid (EDTA), 1×complete protease inhibitior cocktail (EDTA-free; Roche) and 1 mM Pefabloc (Roche), and homogenized on ice using a Potter-Elvehjem homogenizer. The homogenate was centrifuged twice at 10,000x*g* for 5 min at 4°C to remove debris, and then further centrifuged at 100,000x*g* for 60 min at 4°C. The supernatant was collected, not including the floating lipid layer, as the cytosolic extract for the ENGase activity assay. The amount of protein was determined using a Bradford assay (Bio-Rad Protein Assay, Bio-Rad) with bovine serum albumin as the standard.

### ENGase assay

The ENGase activity assay reaction (total 10 μL) included 2 μL of cytosolic fraction in which the protein concentration was adjusted to 30 mg/mL), 4 pmol of Man_9_GlcNAc_2_-PA (TaKaRa Bio Inc.) in a final 50 mM of Mes-NaOH buffer (pH6.0), and the reaction was carried at 30°C for 1 hour. The reaction was terminated by adding 100 μL 75% (vol/vol) ethanol and the resulting solution was incubated on ice for 15 min. After centrifugation at 15,000×*g* for 10 min at 4°C, the supernatant was evaporated to dryness and dissolved in 40 μL of water, and was subjected to HPLC analysis using a Shodex NH2P-50 4E column as described previously [43].

## Supporting information

S1 Fig Product ion spectra of glycopeptides listed in Table 4. **(A-R)** Product ion spectruca of glycopeptides derived from Acid ceramidase (A), Cathepsin D (B), Fibronectin (C-E), Febulin-2 (F, G),Follistatin-related protein 1 (H), Hypoxia up-regulated protein 1 (I), Lysosomal alpha-glucosidase (J), Lysosomal-associated membrane glycoprotein 2 (K), *N*-acetylglucosamine-6-sulfatase (L, M), Peptidyl-prolyl cis-trans isomerase FKBP10 (N, O), Prosaposin (P, Q), and Thrombospondin-1 (R). *Square: *N*-acetylhexosamine, circle: hexose, triangle: deoxyhexose.
(TIF)

S2 Fig *O*-GlcNAc modification is not different among *Ngly1*^*+/+*^, *Ngly1*^*−/−*^, *Ngly1*^*−/−*^*;Engase*^*−/−*^,MEF cells. **(A, B)** Cell lysates form MEF cells were subjected to western blotting using anti-*O*-GlcNAc antibody (CTD110.6, Biolegend). Left and right panels show the result of western blotting and amido black staining of the same membrane, respectively. In panel B, membrane was treated with 50 mM NaOH at 45°C for 19 h (β-elimination) to remove the *O*-GlcNAc modification. Representative data were shown (n = 3).
(TIF)

S3 Fig Confirmation of cell fractionation using subsequent detergent extraction. Extracted cytoplasmic fraction and membrane fraction were subjected to western blotting using anti-LAMP1 (Abcam, ab24170) (A), anti-PDI (Cell Signaling Technology, #2446S), and anti-GAPDH (Millipore, MAB374). Representable data were shown (n = 3).
(TIF)

S1 Movie Behavioral observations of *Ngly1*^*+/+*^*;Engase*^*−/−*^ mice (8 months of age). (MP4)

S2 Movie Behavioral observations of *Ngly1*^*−/−*^*;Engase*^*−/−*^ mice (8 months of age). This mouse is a littermate of the mouse shown in S1 Movie. This mouse showed trembling, a bent spine and coarse fur.
(MP4)

S3 Movie Behavioral observations of *Ngly1*^*+/+*^*;Engase*^*−/−*^ mice (left) and *Ngly1*^*−/−*^*;Engase*^*−/−*^ mice (right). They are littermates and 8 months of age. *Ngly1*^*−/−*^*;Engase*^*−/−*^ mice showing hind-limb clasping and front-limb shaking.
(MP4)

S1 Table Sequence of primers used in this study. Each primer set number corresponds to the number in Fig 1B and 1C.
(DOCX)

S2 Table List of peptides detected as the potential *N*-glycopeptides after PNGase F-digestion. ***N*** in the peptide sequence indicates the potential *N-*glycosylation sites.
(DOCX)

S3 Table List of peptides detected as deamidated peptides in both PNGase F-treated and -untreated samples. ***n*** in the peptide sequence indicates the sites most likely deamidated in a PNGase F digestion-independent manner.
(DOCX)

S4 Table Comparison of phenotypes of *Ngly1*^*−/−*^*;Engase*^*−/−*^ mice in C57BL/6 background or *Ngly1*^*−/−*^ mice in C57BL/6 and ICR mixed background with symptoms of *NGLY1*-deficiency subjects. Description of symptoms is according to ref. 24.
(DOCX)
