# Supplementary figures and images for: Lethality of mice bearing a knockout of the Ngly1-gene is partially rescued by the additional deletion of the Engase gene
Source: PLoS Genet. 2017 Apr 20;13(4):e1006696. doi: 10.1371/journal.pgen.1006696 (PMC5398483; doi:10.1371/journal.pgen.1006696)

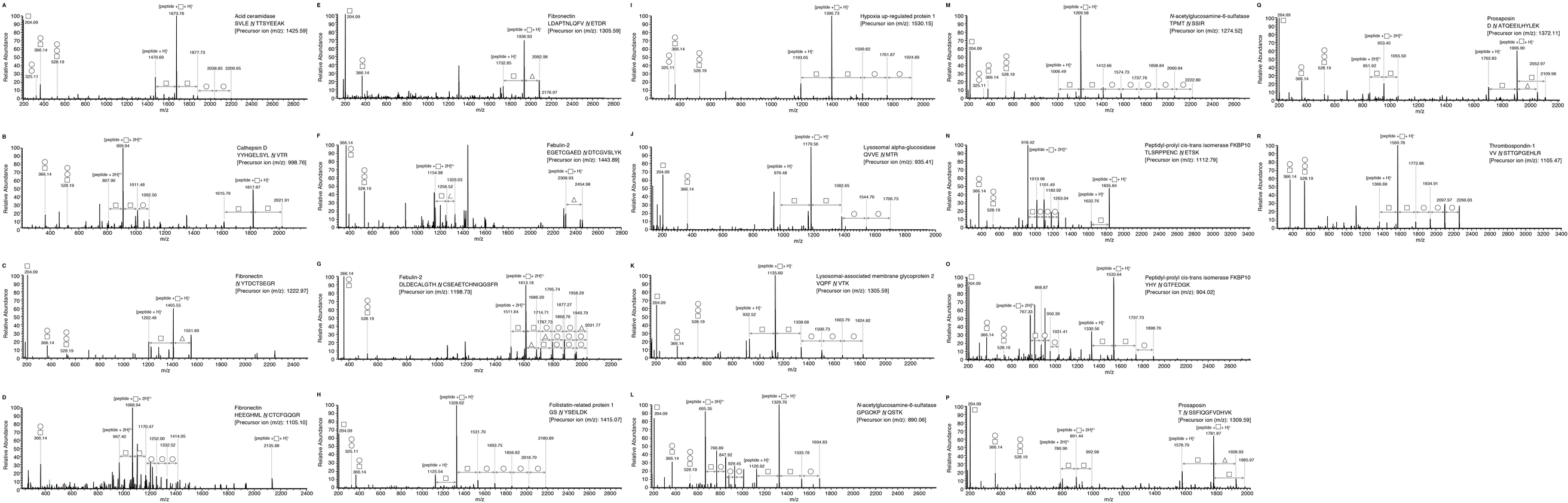

Supplement: S1 Fig — (A-R) Product ion spectruca of glycopeptides derived from Acid ceramidase (A), Cathepsin D (B), Fibronectin (C-E), Febulin-2 (F, G),Follistatin-related protein 1 (H), Hypoxia up-regulated protein 1 (I), Lysosomal alpha-glucosidase (J), Lysosomal-associated membrane glycoprotein 2 (K), N-acetylglucosamine-6-sulfatase (L, M), Peptidyl-prolyl cis-trans isomerase FKBP10 (N, O), Prosaposin (P, Q), and Thrombospondin-1 (R). *Square: N-acetylhexosamine, circle: hexose, triangle: deoxyhexose. (TIF) [file pgen.1006696.s001.tif]

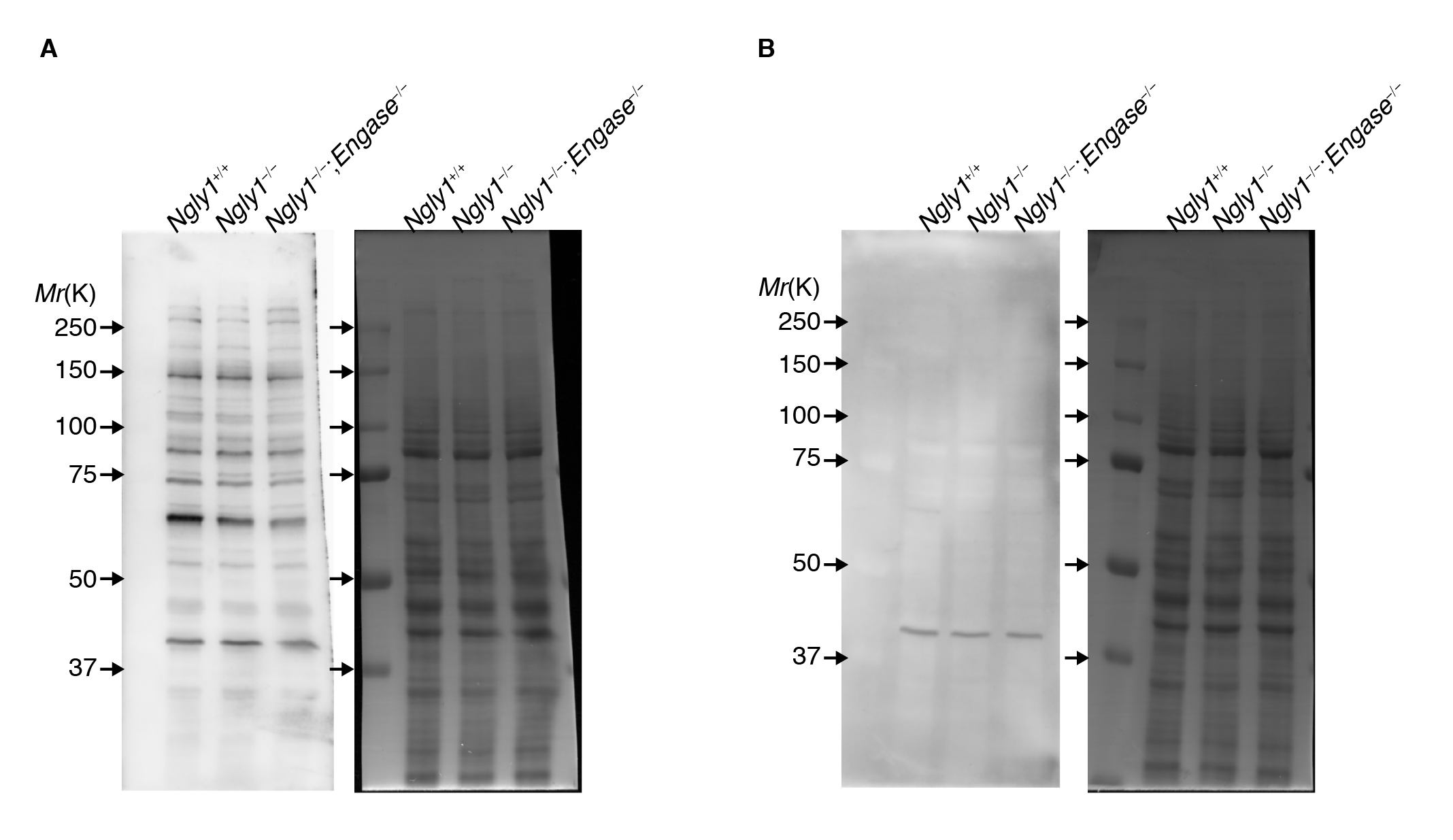

Supplement: S2 Fig — (A, B) Cell lysates form MEF cells were subjected to western blotting using anti-O-GlcNAc antibody (CTD110.6, Biolegend). Left and right panels show the result of western blotting and amido black staining of the same membrane, respectively. In panel B, membrane was treated with 50 mM NaOH at 45°C for 19 h (β-elimination) to remove the O-GlcNAc modification. Representative data were shown (n = 3). (TIF) [file pgen.1006696.s002.tif]

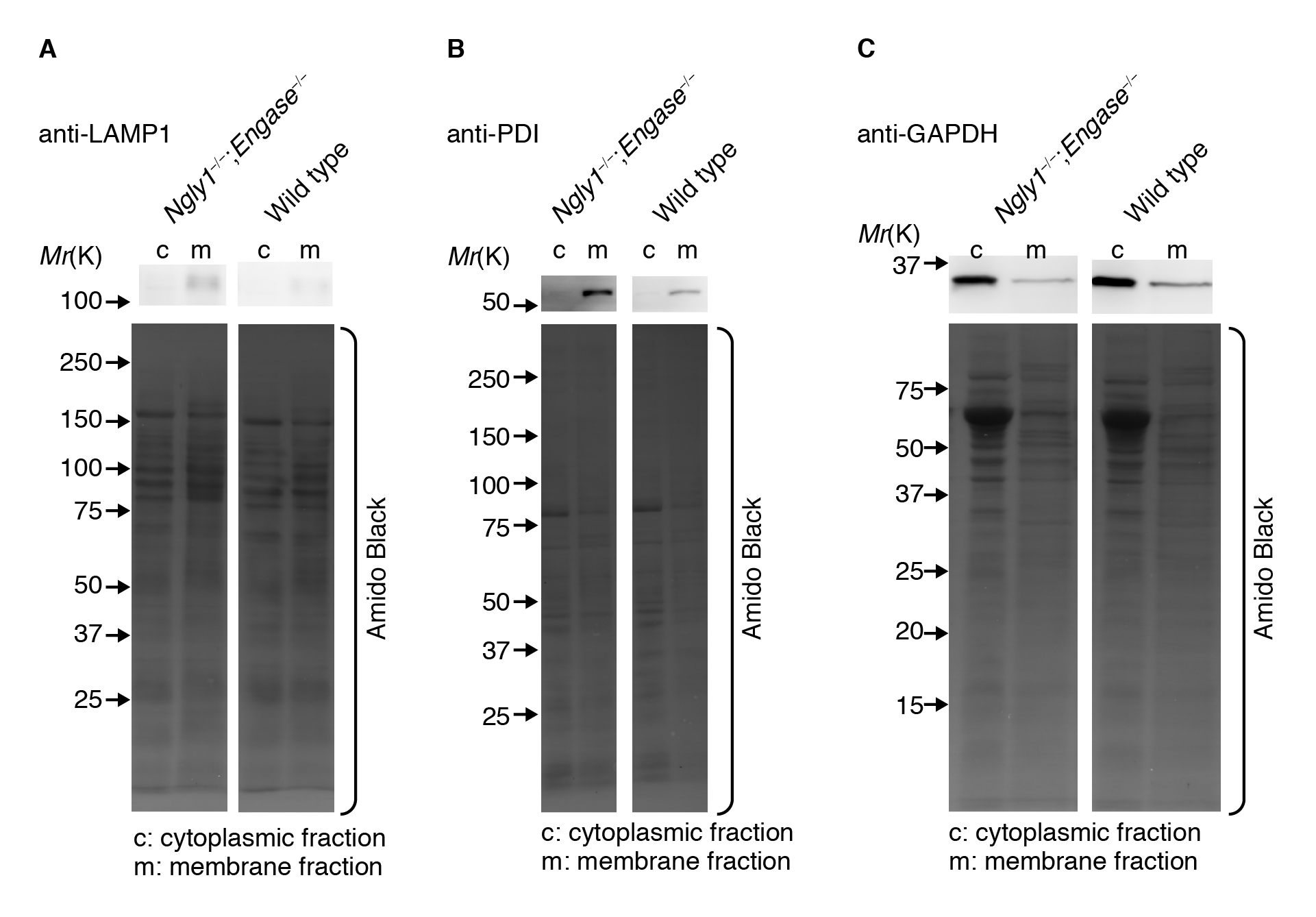

Supplement: S3 Fig — Extracted cytoplasmic fraction and membrane fraction were subjected to western blotting using anti-LAMP1 (Abcam, ab24170) (A), anti-PDI (Cell Signaling Technology, #2446S), and anti-GAPDH (Millipore, MAB374). Representable data were shown (n = 3). (TIF) [file pgen.1006696.s003.tif]
