# Supplementary material for: Lethality of mice bearing a knockout of the Ngly1-gene is partially rescued by the additional deletion of the Engase gene
Source: PLoS Genet. 2017 Apr 20;13(4):e1006696. doi: 10.1371/journal.pgen.1006696 (PMC5398483; doi:10.1371/journal.pgen.1006696)
Supplement: S1 Table — Each primer set number corresponds to the number in Fig 1B and 1C. (DOCX) [file pgen.1006696.s007.docx]

**Supplemental Table 1 | Sequences of primers used in this study.**

| **Primer set #** | **Sequence** | **Band size (bp)** |
| --- | --- | --- |
| 1 | CCTGTGGCATCACTGTCATC | 477 |
|  | ACTTGTGCGGATGGAAACAG |  |
| 2 | CCAGTAAATAGTAGGCCAAC | 760 |
|  | AGCTGGACTTTGTAGTGG |  |
| 3 | CTCTTCTCATACAGACACAG | 422 |
|  | GAATATGATCGGAATTGGGCTG |  |
| 4 | CAGCCCAATTCCGATCATATTC | 337 |
|  | TTGGATGGGTCAAGACTAGC |  |
| 5 | TGGTTAGCGTACGCATTGGG | 615 |
|  | CCACTGAAACAAACGCTCACAG |  |
| 6 | TGACGGCTGGCTCATCAACATCG | 522 |
|  | GGAGATGAGACCGTTGCGAG |  |
| 7 | TGGTTAGCGTACGCATTGGG | 717 |
|  | TGAGCCCAGAAAGCGAAGGAGC |  |
| 8 | GGGTGGGGTGGGATTAGA | 766 |
|  | CTGATGGAGCTGTGCGGTAAGGT |  |

* Primer sets used were same as the ones previous reported [27].
