# Supplementary material for: Lethality of mice bearing a knockout of the Ngly1-gene is partially rescued by the additional deletion of the Engase gene
Source: PLoS Genet. 2017 Apr 20;13(4):e1006696. doi: 10.1371/journal.pgen.1006696 (PMC5398483; doi:10.1371/journal.pgen.1006696)
Supplement: S2 Table — N in the peptide sequence indicates the potential N-glycosylation sites. (DOCX) [file pgen.1006696.s008.docx]

**Supplemental Table 2 | List of peptides detected as the potential *N­-*glycopeptides after PNGase F-digestion**

| Protein name | Peptide Sequence* | [peptide + H]+ |
| --- | --- | --- |
| Cysteine and glycine-rich protein 1 | HEEAPGHRPTTNP ***N*** ASK | 1843.87 |
| MLV-related proviral Env polyprotein | THQALC ***N*** TTQK | 1302.61 |
| Protein S100-A11 | DG ***N*** NTQLSK | 977.45 |
| Vinculin | AANFE ***N*** HSGR | 1103.48 |
| *N*-acetylglucosamine-6-sulfatase | GPGIKP ***N*** QTSK | 1127.61 |
| Tetraspanin-3 | TY ***N*** GTNSDAASR | 1257.53 |
| Peptidyl-prolyl cis-trans isomerase  FKBP10 | TLSRPPENC ***N*** ETSK | 1633.75 |
| Legumain | SHT ***N*** TSHVMQYG ***N*** K | 1605.70 |
| Fibronectin | ***N*** YTDCTSEGR | 1203.46 |
| Thrombospondin-1 | VV ***N*** STTGPGEHLR | 1367.69 |
| *N*-acetylglucosamine-6-sulfatase | TPMT ***N*** SSIR | 1007.48 |
| Lysosomal alpha-glucosidase | QVVE ***N*** MTR | 977.47 |
| Hypoxia up-regulated protein 1 | E ***N*** GTDAVQEEEESPAEGSK | 2006.83 |
| Peptidyl-prolyl cis-trans isomerase  FKBP10 | YHY ***N*** GTFEDGK | 1331.55 |
| 60S ribosomal protein L3 | N ***N*** ASTDYDLSDK | 1343.56 |
| Fibronectin | HEEGHML ***N*** CTCFGQGR | 1933.77 |
| Glucosylceramidase | DLGPALA ***N*** SSHDVK | 1424.70 |
| Transgelin-2 | ***N*** FSDNQLQEGK | 1280.58 |
| Lysosome-associated membrane  glycoprotein 2 | VQPF ***N*** VTK | 933.51 |
| Phosphoribosyl pyrophosphate synthase-  associated protein 1 | VFSA ***N*** STAACTELAK | 1570.75 |
| Acid ceramidase | SVLE ***N*** TTSYEEAK | 1471.68 |
| Myosin light polypeptide 6 | EG ***N*** GTVMGAELR | 1234.57 |
| Heat shock protein HSP 90-alpha | ELIS ***N*** SSDALDK | 1292.62 |
| Fibulin-2 | EGETCGAED ***N*** DTCGVSLYK | 2105.83 |
| Follistatin-related protein 1 | GS ***N*** YSEILDK | 1126.53 |
| Hypoxia up-regulated protein 1 | VFGSQ ***N*** LTTVK | 1194.64 |
| Fibulin-2 | DLDECALGTH ***N*** CSEAETCHNIQGSFR | 3022.24 |
| Talin-1 | DDIL ***N*** GSHPVSFDK | 1544.72 |
| Thrombospondin-1 | VSCPIMPCS ***N*** ATVPDGECCPR | 2407.98 |
| Carboxypeptidase N subunit 2 | LQLL ***N*** LSR | 957.57 |
| C-type mannose receptor 2 | WNDSPC ***N*** QSLPSICK | 1806.78 |
| Cathepsin D | YYHGELSYL ***N*** VTR | 1615.77 |
| Lysosomal alpha-glucosidase | LE ***N*** LSSTESGYTATLTR | 1843.89 |
| Prolow-density lipoprotein receptor-  related protein 1 | IETILL ***N*** GTDR | 1245.67 |
| Lysosomal protective protein | MYVT ***N*** DTEVAENNYEALK | 2104.94 |
| Putative phospholipase B-like 2 | SDLNPA ***N*** GSYPFQALHQR | 2015.96 |
| Fibulin-2 | ACHCPDAGGELICYQLPGCHG ***N*** FSDAEEGDSER | 3709.46 |
| Mesothelin | YVSPEDIHQW ***N*** VTSPDTVK | 2216.06 |
| Elongation factor 1-alpha 2 | VETGILRPGMVVTFAPV ***N*** ITTEVK | 2572.41 |
| Fibronectin | LDAPTNLQFV ***N*** ETDR | 1733.83 |
| Dihydropyrimidinase-related protein 2 | DNFTLIPEGT ***N*** GTEER | 1793.82 |
| Endoplasmin | HN ***N*** DTQHIWESDSNEFSVIADPR | 2712.19 |
| Lumican | LHINYN ***N*** LTESVGPLPK | 1910.00 |
| Lysosomal alpha-glucosidase | GVFIT ***N*** ETGQPLIGK | 1574.84 |
| Lysosome-associated membrane  glycoprotein 1 | E ***N*** VSDPSLTITFGR | 1536.76 |
| CD63 antigen | ENNCCGAS ***N*** YTDWENIPGMAK | 2431.96 |
| Cathepsin L1 | AEFAVA ***N*** DTGFVDIPQQEK | 2079.99 |
| Prosaposin | T ***N*** SSFIQGFVDHVK | 1579.78 |
| Lysosome-associated membrane  glycoprotein 2 | EASHYSIHDIVLSY ***N*** TSDSTVFPGAVAK | 3009.45 |
| Calumenin | ***N*** ATYGYVLDDPDPDDGFNYK | 2279.96 |
| Prosaposin | D ***N*** ATQEEILHYLEK | 1703.81 |
| Transmembrane glycoprotein NMB | ***N*** LSDEIFLR | 1107.57 |
| Lysosome-associated membrane  glycoprotein 1 | AFNISP ***N*** DTSSGSCGINLVTLK | 2296.11 |
| Alpha-actinin-4 | ISIEM ***N*** GTLEDQLSHLK | 1928.97 |
| Peptidyl-prolyl cis-trans isomerase  FKBP9 | YHY ***N*** GTLLDGTLFDSSYSR | 2210.00 |
| Protein deglycase DJ-1 | TQGPYDVVVLPGGnLGAQ ***N*** LSESPMVK | 2772.37 |
| Lysosome-associated membrane  glycoprotein 1 | GYLLTL ***N*** FTK | 1170.64 |
| Palladin | SPVDESGDEVQDPDVPVE ***N*** ATAPFFEMK | 3050.34 |
| Transferrin receptor protein 1 | ***N*** ITAF ***N*** ETLFR | 1327.65 |
| Biglycan | MIE ***N*** GSLSFLPTLR | 1578.82 |
| Putative phospholipase B-like 2 | LEDGFHPDAVAWA ***N*** LTNAIR | 2211.08 |
| Receptor-type tyrosine-protein  phosphatase kappa | GPLANPIW ***N*** VTGFTGR | 1700.88 |
| Alpha-2-HS-glycoprotein | RPFGVVYEMEVDTLETTCHALDPTPLA ***N*** CSVR | 3694.72 |
| ATP-binding cassette sub-family A  member 13 | QVDMIIT ***N*** LTEDLNVASQSNWKHFK | 2932.46 |
| Lysosomal alpha-glucosidase | EVTVLGVATAPTQVLSNGIPVS ***N*** FTYSPDNK | 3219.65 |
| *N*-acetylglucosamine-6-sulfatase | MLVSNIDLGPTILDLAGYDL ***N*** K | 2376.23 |

****N*: glycosylation site**
