# Supplementary material for: Lethality of mice bearing a knockout of the Ngly1-gene is partially rescued by the additional deletion of the Engase gene
Source: PLoS Genet. 2017 Apr 20;13(4):e1006696. doi: 10.1371/journal.pgen.1006696 (PMC5398483; doi:10.1371/journal.pgen.1006696)
Supplement: S3 Table — n in the peptide sequence indicates the sites most likely deamidated in a PNGase F digestion-independent manner. (DOCX) [file pgen.1006696.s009.docx]

**Supplemental Table 3 | List of peptides detected as deamidated peptides in both PNGase F-treated and –untreated samples**

| Protein name | Peptide Sequence* | [peptide + H]+ |
| --- | --- | --- |
| Heat shock cognate  71 kDa protein | ***n*** QTAEKEEFEHQQK | 1746.79 |
| Myristoylated alanine-rich  C-kinase substrate | EELQA ***n*** GSAPAADK | 1401.65 |
| Protein deglycase DJ-1 | MM ***n*** GSHYSYSESR | 1549.61 |
| Myristoylated alanine-rich  C-kinase substrate | EELQA ***n*** GSAPAADKEEPASGSAATPAAAEK | 2869.33 |
| Myosin-9 | DELADEIA ***n*** SSGK | 1349.61 |
| MARCKS-related protein | GEGESPPV ***n*** GTDEAAGATGDAIEPAPPSQEAEAK | 3250.45 |
| Ubiquitin-like modifier-  activating enzyme 1 | ***n*** GSEADIDESLYSR | 1556.67 |
| Dual specificity mitogen-activated  protein kinase kinase 1 | KPTPIQLNPAPDGSAV ***n*** GTSSAETNLEALQK | 3149.60 |
| Eukaryotic translation  initiation factor 1 | FAC ***n*** GTVIEHPEYGEVIQLQGDQR | 2761.29 |
| 60S ribosomal protein L5 | N ***n*** VTPDMMEEMYK | 1602.65 |
| Serine/threonine-protein kinase PAK 2 | ALYLIAT ***n*** GTPELQNPEK | 1973.02 |
| Tubulin beta | ***n*** SSYFVEWIPNNVK | 1697.82 |
| NEDD8-activating enzyme  E1 regulatory subunit | NVNTAL ***n*** TTQIPSSIEDIFNDDR | 2578.24 |
|  |  |  |

*** *n*: deamidated site**
