# Supplementary material for: Lethality of mice bearing a knockout of the Ngly1-gene is partially rescued by the additional deletion of the Engase gene
Source: PLoS Genet. 2017 Apr 20;13(4):e1006696. doi: 10.1371/journal.pgen.1006696 (PMC5398483; doi:10.1371/journal.pgen.1006696)
Supplement: S4 Table — Description of symptoms is according to ref. 24. (DOCX) [file pgen.1006696.s010.docx]

**Supplemental Table 4 | Comparison of phenotypes of *Ngly1^−/−^;Engase^−/−^* mice in C57BL/6 background or *Ngly1^−/−^* mice in C57BL/6 and ICR mixed background with symptoms of *NGLY1*-deficiency subjects.**

| **Mice Phenotypes** | **Symptoms of *NGLY1* genetic disorders (24)** |
| --- | --- |
| Small body size | Developmental delay |
| Trembling, Hind-limb clasping | Movement disorder |
| Eye opacity | Hypo/alacrima |
| Bent spine | Scoliosis |
